# Mechanism Through Which Antioxidant Polysaccharide from *Tetrastigma hemsleyanum* Protects Against DSS-Induced Ulcerative Colitis: Insights from Multi-Omics

**DOI:** 10.3390/molecules31111974

**Published:** 2026-06-05

**Authors:** Ling Zhang, Wei Xu, Xinyu Liao, Guoqi Yuan, Chen Jin, Huan Xiao, Huabin Liu, Zhitong Jin, Yaqiong Deng, Yang Liu

**Affiliations:** 1 School of Pharmacy, Jiangxi University of Chinese Medicine, Nanchang 330004, China; 2 School of Pharmacy, Nanchang Medical College, Nanchang 330052, China; 3 Department of Natural Medicine, School of Pharmacy, Fudan University, Shanghai 201203, China; 4 Jiangxi Provincial Administration of Chinese Medicine Key Laboratory for Anti-inflammatory Chinese Medicine Efficacy and Quality Control, Nanchang 330052, China

**Keywords:** *Tetrastigma hemsleyanum* polysaccharide, ulcerative colitis, gut microbiota, PI3K/AKT signaling pathway

## Abstract

*Tetrastigma hemsleyanum* polysaccharide (TH-P) exhibited anti-inflammatory and intestinal protective activities, but its mechanism against ulcerative colitis (UC) remained unclear. This study used a multi-omics approach to elucidate the effects of TH-P in protecting against dextran sulfate sodium (DSS)-induced UC mice and the underlying mechanisms. In vitro, TH-P dose-dependently suppressed LPS-induced ROS production and pro-inflammatory cytokine release in RAW264.7 cells. In vivo, TH-P alleviated DSS-induced weight loss, disease activity index, colon shortening, edema, and mucosal damage. Transcriptomic analysis and Western blotting revealed that TH-P significantly reversed DSS-induced gene expression alterations, with particular enrichment of the PI3K/AKT signaling pathway. Serum metabolomics showed that TH-P restored metabolic disturbances in glycerophospholipid and arachidonic acid metabolism. The results of 16S rRNA sequencing indicated that TH-P increased microbial diversity, enriched beneficial *Bacteroidota*, and reduced opportunistic *Actinomycetota* and *Pseudomonadota*. Untargeted metabolomics further demonstrated elevated acetate, propionate, and butyrate levels. Collectively, TH-P alleviated UC through a multi-target mechanism involving antioxidant and anti-inflammatory activities, gut microbiota modulation, enhanced SCFA production, and activation of the PI3K/AKT signaling pathway.

## 1. Introduction

Ulcerative colitis (UC) is a chronic, idiopathic inflammatory bowel disease that primarily affects the colorectum [1,2]. The disease typically begins in the rectum and extends contiguously in a continuous and diffuse manner [3,4]. Its pathological features are mainly characterized by diffuse inflammatory cell infiltration in the mucosal and submucosal layers, distortion of crypt architecture, and disruption of epithelial barrier function [5].

The incidence and prevalence of UC have increased globally in recent years [6]. Currently, clinical treatment of UC primarily relies on aminosalicylates, corticosteroids, immunosuppressants, and biologics [7]. However, these therapeutic strategies have limitations: some patients ultimately require colectomy, and long-term medication use is associated with decreased efficacy, opportunistic infections, and increased risk of malignancy [8]. Therefore, further investigation into the pathogenesis of UC and the development of novel, highly effective, and low-toxicity therapeutic strategies remain critical in this field.

Plant polysaccharides, a class of natural macromolecules with diverse bioactivities, have attracted considerable attention due to their low toxicity, high safety profile, and multi-target effects [9,10]. Numerous studies have demonstrated that plant polysaccharides exert anti-inflammatory and intestinal protective effects through multiple mechanisms, including modulation of gut microbiota composition, promotion of short-chain fatty acid (SCFA) production, maintenance of intestinal barrier integrity, and regulation of inflammatory signaling pathways [11,12,13]. For example, *Dendrobium officinale* polysaccharide has been reported to ameliorate DSS-induced UC by modulating gut microbiota and promoting SCFA production, thereby activating the PI3K/AKT pathway [14]. Similarly, *Astragalus membranaceus* polysaccharide alleviates experimental colitis by regulating intestinal microbiota and the PI3K/AKT signaling pathway, while *Polygonatum sibiricum* polysaccharide improves UC via gut-microbiota-mediated SCFA metabolism and PI3K/AKT activation [15]. These findings suggest that the “polysaccharide-gut microbiota-SCFAs-PI3K/AKT pathway” axis represents a common mechanism that underlies the intestinal protective effects of plant polysaccharides.

The PI3K/AKT signaling pathway is a critical intracellular pathway that regulates cell survival, proliferation, differentiation, apoptosis, and inflammatory responses [16,17]. In UC, dysregulation of the PI3K/AKT pathway is closely associated with intestinal inflammation and mucosal barrier dysfunction [18]. Activation of this pathway has been shown to promote tight junction protein expression, enhance intestinal barrier integrity, and suppress inflammatory responses [19,20]. Several plant polysaccharides have been reported to alleviate intestinal inflammation.

*Tetrastigma hemsleyanum* Diels, a plant belonging to the *Vitaceae* family, contains a polysaccharide that has been demonstrated to possess anti-inflammatory and antioxidant activities [21]. However, the protective effects of TH-P against UC and the protective mechanism of TH-P have not been fully explored. Through an integrated multi-omics approach, this study revealed that TH-P alleviated UC by activating the PI3K/AKT pathway, remodeling lipid metabolism, modulating gut microbiota (enriching *Bacteroidota* and reducing *Actinomycetota/Pseudomonadota*) and suppressing LPS-induced ROS/cytokines in vitro, thereby establishing a multi-target network.

## 2. Results

### 2.1. Analysis of Content and Structural Characteristics of TH-P

**Figure 1:**
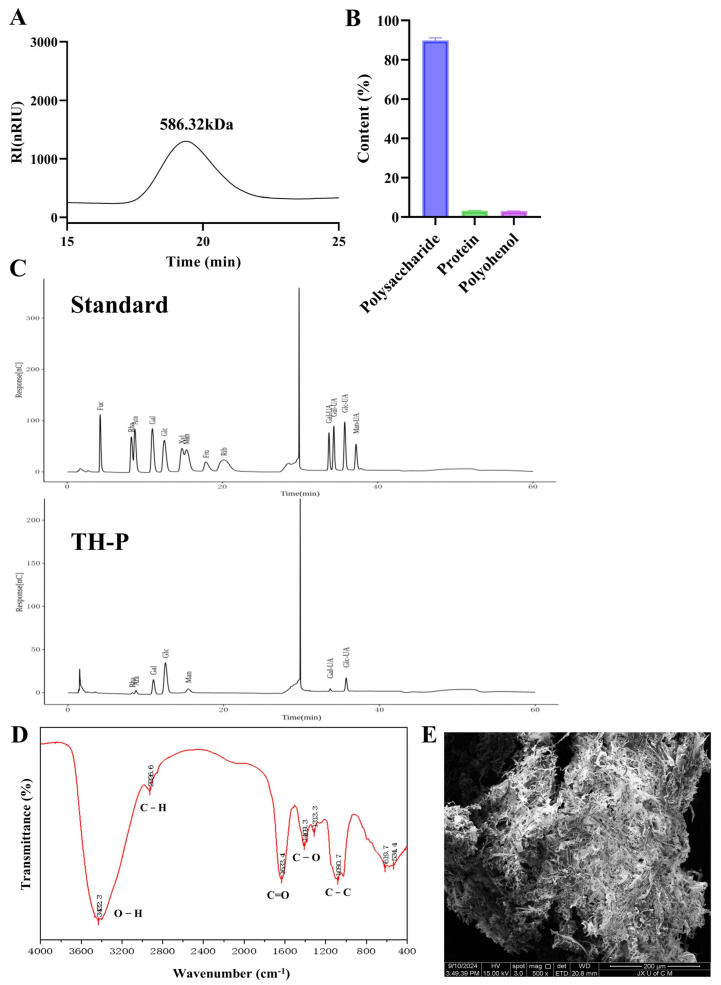
The fundamental structural characteristics of TH-P. (**A**) Molecular weight; (**B**) total polysaccharide, glucuronic acid, polyphenol and protein contents; (**C**) monosaccharide composition: rhamnose (Rha), arabinose (Ara), galactose (Gal), glucose (Glc), mannose (Man), galacturonic acid (Gla-UA), and glucuronic acid (Glu-UA); (**D**) FT-IR analysis; (**E**) SEM results of TH-P.

TH-P primarily consisted of fragments with a molecular weight of 55.47 kDa (Figure 1A). The molecular parameters, chromatogram plot, distribution plot and the standard of HPGPC of TH-P were showed in Table S1, Figures S1 and S2. The total polysaccharide content was 69.1%, with the glucuronic acid, protein, and polyphenol contents at 21.6%, 3.1%, and 3.0%, respectively (Figure 1B). TH-P was primarily composed of seven monosaccharides: rhamnose, arabinose, galactose, glucose, mannose, galacturonic acid, and glucuronic acid, with a molar ratio of 4.49: 1.76: 15.18: 45.84: 8.59: 4.5: 19.61 (Figure 1C). Moreover, the Figure S3 showed the standard of monosaccharide composition about TH-P. The infrared spectroscopic analysis of THP revealed that the strong absorption peak around 3432.3 cm^−1^ corresponded to the stretching vibration of the hydroxyl groups (–OH), which were most abundant in the sugar chains; the absorption peak at 2926.6 cm^−1^ was attributed to C-H stretching vibration; the more pronounced absorption near 1633.4 cm^−1^ originated from the C=O stretching vibration in the –COOH groups; and the absorption peak at 1409.3 cm^−1^ was caused by the angular vibration of the C-H bonds. The weak absorption peak at 1080.7 cm^−1^ was attributed to the stretching vibrations of the C-O and C-C bonds in the pyranose ring, indicating the presence of α-pyranose in the TH-P (Figure 1D). The infrared spectrum of the TH-P exhibited typical polysaccharide characteristics. SEM is more commonly applied to directly observe the microstructures and aggregation properties of polysaccharides. The morphological picture (Figure 1E) shows that the macroaggregated state of TH-P presented as irregularly curled sheets with smooth surfaces.

### 2.2. Antioxidant and Anti-Inflammatory Effects of TH-P In Vitro

**Figure 2:**
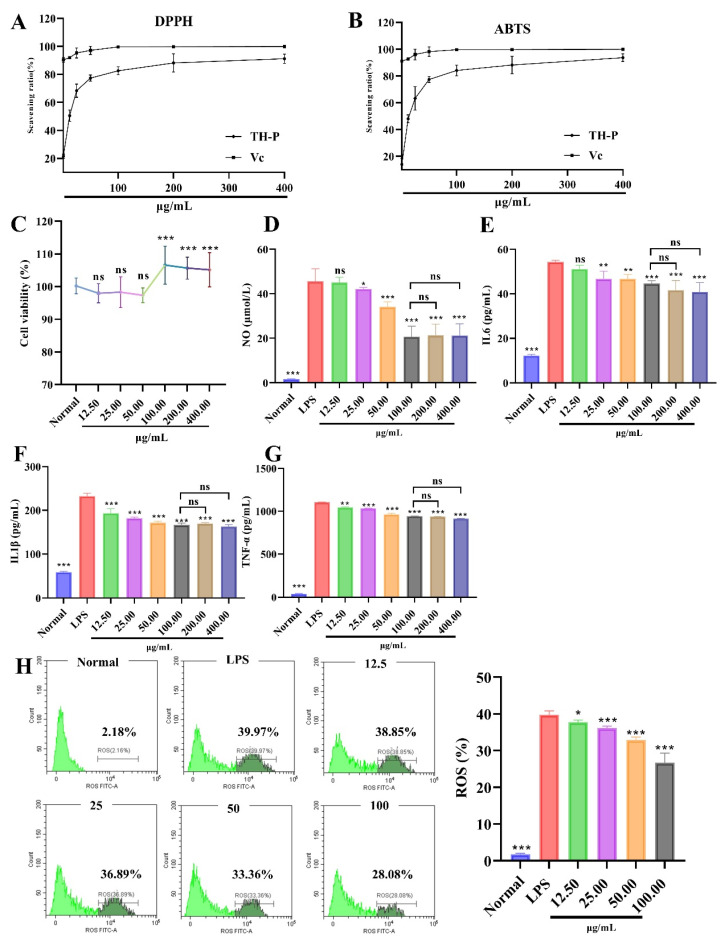
In vitro antioxidant and anti-inflammatory effects of TH-P. (**A**) DPPH and (**B**) ABTS radical scavenging activity; effect of TH-P on (**C**) cell viability, (**D**) NO release, (**E**) IL-6 level, (**F**) IL-1β level and (**G**) TNF-α level in LPS-induced RAW 264.7 cells. (**H**) Flow cytometry analysis and quantification of ROS levels in LPS-induced RAW 264.7 cells treated with TH-P. Data are presented as mean ± SD (*n* = 4); * *p* < 0.05, ** *p* < 0.01, *** *p* < 0.001, compared with LPS group; ns, compared with 100 group.

TH-P exhibited a dose-dependent increase in DPPH and ABTS radical scavenging activity, indicating strong antioxidant capacity (Figure 2A,B). Moreover, TH-P showed no significant cytotoxicity toward RAW264.7 macrophages within the tested concentration range (Figure 2C). LPS stimulation markedly increased NO production, whereas TH-P significantly inhibited NO release in a dose-dependent manner (Figure 2D). TH-P significantly reduced the secretion of IL-6, IL-1β, and TNF-α (Figure 2E–G) as well. Notably, TH-P markedly suppressed ROS accumulation (Figure 2H). TH-P exhibited notable antioxidant activity primarily due to its high uronic acid content (providing carboxyl groups for metal chelation and electron donation) as well as the presence of galactose and arabinose (associated with enhanced radical scavenging) [22]. These results demonstrated that TH-P exerted potent antioxidant and anti-inflammatory effects in vitro.

### 2.3. Ameliorative Effects of TH-P on DSS-Induced UC in Mice

**Figure 3:**
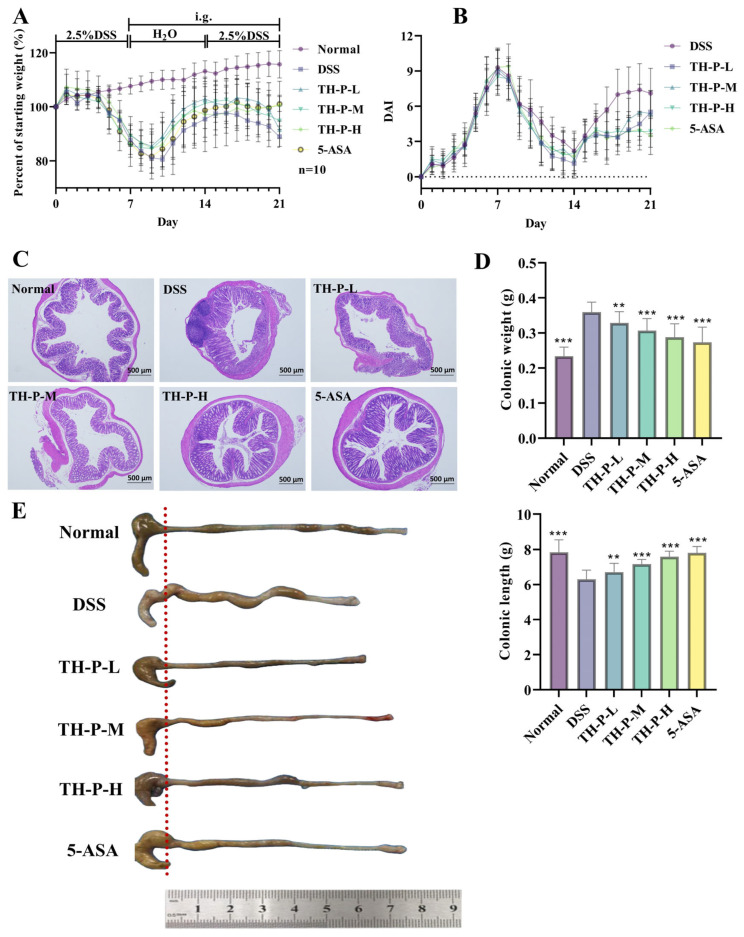
Protective effects of TH-P on pathological damage in DSS-induced mice with UC. (**A**) Body weight change curves, (**B**) disease activity index (DAI) score change curve in DSS-induced mice with UC. (**C**) Colonic pathological change (scale bar: 500 μm), (**D**) colon weight change and (**E**) colon length change in colon of UC mice. Data are presented as mean ± SD (*n* = 10); ** *p* < 0.01, *** *p* < 0.001 compared with DSS group.

DSS treatment induced significant weight loss, whereas TH-P markedly alleviated this effect, with the high-dose group showing the most pronounced recovery (Figure 3A). DAI scores were significantly increased in DSS-treated mice and were effectively reduced by TH-P (Figure 3B). Severe mucosal damage, crypt disruption, and inflammatory infiltration were observed in the DSS group, whereas TH-P treatment markedly restored epithelial integrity and reduced inflammation (Figure 3C). DSS significantly increased colon weight and shortened colon length, whereas TH-P reversed these pathological changes (Figure 3D,E). These findings indicate that TH-P effectively attenuated DSS-induced colonic injury.

### 2.4. Effects of TH-P on Inflammatory Cytokines and Gene Expression

**Figure 4:**
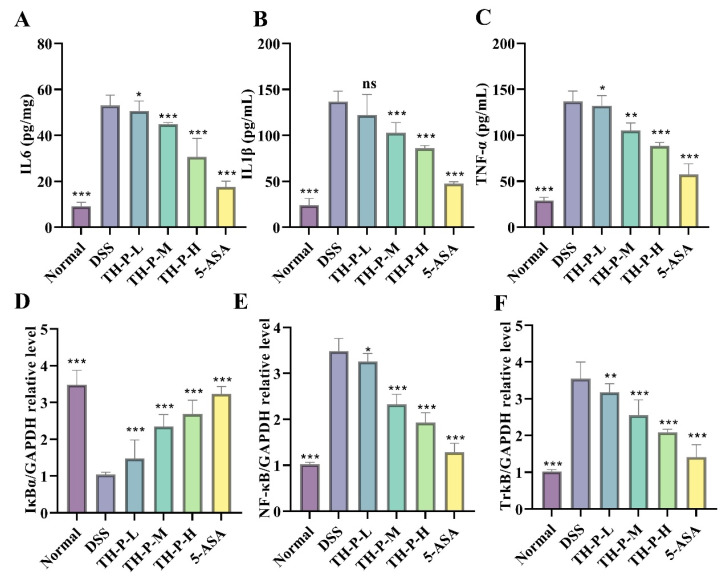
Regulatory effects of TH-P on inflammatory factors and related gene expression in colon tissue of DSS-induced mice with UC (*n* = 6). (**A**–**C**) Detection of IL-6, IL-1β and TNF-α levels in colon tissue; (**D**–**F**) relative mRNA expression of IκBα, NF-κB and TrkB in colon tissue. * *p* < 0.05, ** *p* < 0.01, *** *p* < 0.001 compared with DSS group.

DSS significantly increased IL-6, IL-1β, and TNF-α levels, whereas TH-P treatment markedly reduced these pro-inflammatory cytokines (Figure 4A–C). At the RNA level, DSS suppressed the mRNA level of IκBα and increased NF-*κ*b and TrκB expression. The disorder was effectively regulated by TH-P (Figure 4D–F). These results suggested that TH-P ameliorated DSS-induced colitis by regulating inflammatory cytokine.

### 2.5. The Transcriptomic Analysis of Colon in Mice with UC

**Figure 5:**
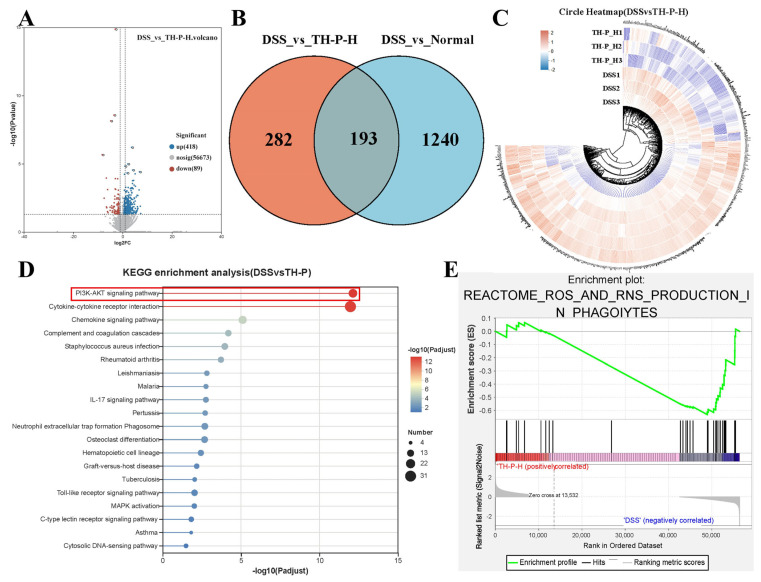
Transcriptomic analysis of colon of mice with UC (*n* = 3). (**A**) Volcano plot, (**B**) Venn plot and (**C**) correlation analysis of colon of mice with UC. (**D**) KEGG enrichment analysis shows enrichment of PI3K/AKT signaling between TH-P group and DSS group. (**E**) Gene set enrichment analysis (GSEA) shows negative association ROS-related gene sets between DSS group and TH-P group.

The volcano plot, Venn plot, and correlation analysis revealed that TH-P treatment significantly altered DSS-induced gene expression profiles (Figure 5A–C). Differentially expressed genes were enriched in pathways related to inflammation, immune regulation, oxidative stress, and apoptosis (Figure 5D), and the PI3K/AKT signaling pathway was enriched. Notably, oxidative-stress-related pathways were significantly enriched according to gene set enrichment analysis (GSEA) (Figure 5E). These results indicated that TH-P exerted protective effects through the regulation of PI3K/AKT signaling and oxidative stress.

### 2.6. Serum Untargeted Metabolomics Analysis of Mice with UC

**Figure 6:**
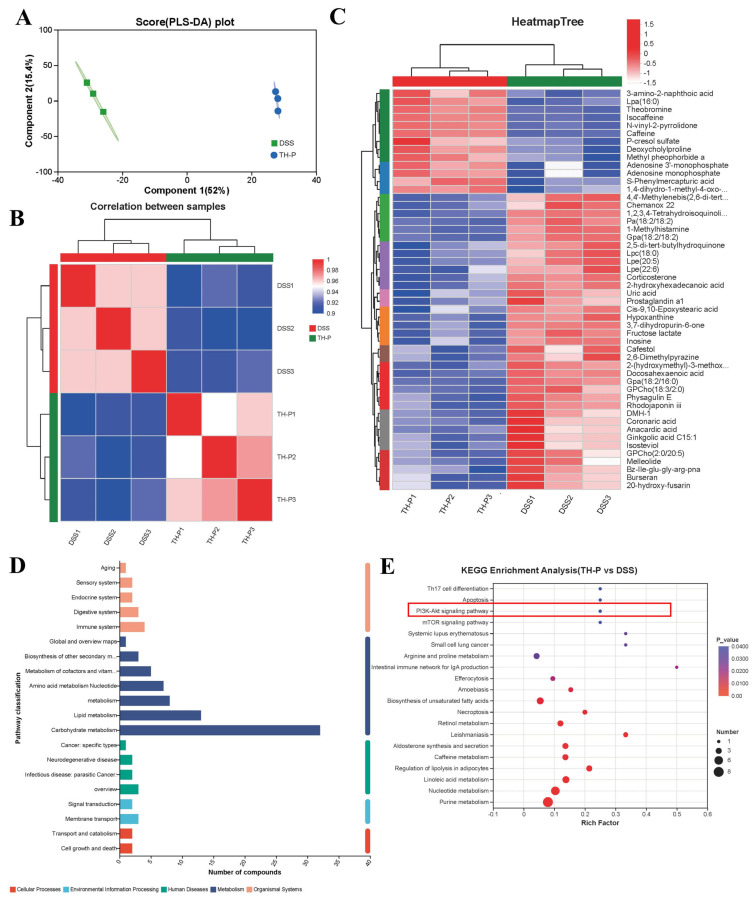
Serum untargeted metabolomics analysis of mice with UC (*n* = 3). (**A**) PLS-DA score plot, (**B**) correlation heatmap, (**C**) clustering of differential metabolites and (**D**) metabolite classification showing difference between TH-P group and DSS group; (**E**) KEGG showing PI3K/AKT signaling was enriched between TH-P group and DSS group.

DSS induced significant alterations in systemic metabolic profiles, whereas TH-P changed metabolic patterns (Figure 6A). The high reproducibility and reliability of the metabolomic data were confirmed (Figure 6B). TH-P markedly reversed DSS-induced metabolic disturbances (Figure 6C). Differential metabolites were mainly involved in amino acid, carbohydrate, and nucleotide metabolism (Figure 6D). Inflammation-related metabolic pathways were significantly enriched (Figure 6E). These results demonstrated that TH-P restored the systemic metabolic homeostasis induced by DSS.

### 2.7. Effects of TH-P on PI3K/AKT Pathway and Oxidative Stress in Mice with UC

**Figure 7:**
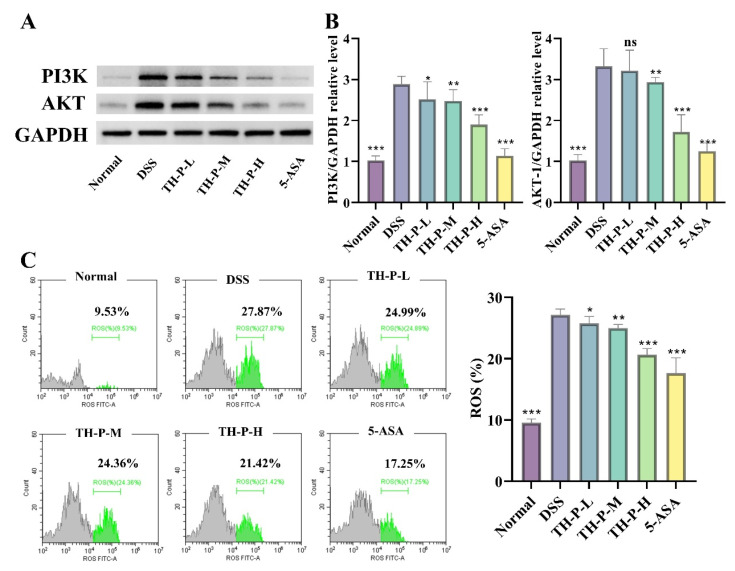
Effects of TH-P on PI3K/AKT pathway and oxidative stress in mice with UC (*n* = 4). (**A**) Western blot analysis of PI3K and AKT; (**B**) Quantitative analysis of protein expression in PI3K and AKT; (**C**) ROS levels and quantitative analysis of ROS in colon tissue. Data are mean ± SD (*n* = 6). * *p* < 0.05, ** *p* < 0.01, *** *p* < 0.001 vs. DSS.

DSS significantly increased PI3K and AKT expression, whereas TH-P suppressed its expression in a dose-dependent manner, indicating the inhibition of the PI3K/AKT pathway (Figure 7A,B). Furthermore, TH-P markedly reduced the ROS accumulation in colon tissues, indicating the effective attenuation of oxidative stress (Figure 7C). In agreement with the transcriptomic data, these results demonstrated that TH-P alleviated UC by inhibiting the PI3K/AKT pathway and reducing ROS accumulation.

### 2.8. Gut Microbiota Analysis in Mice with UC

**Figure 8:**
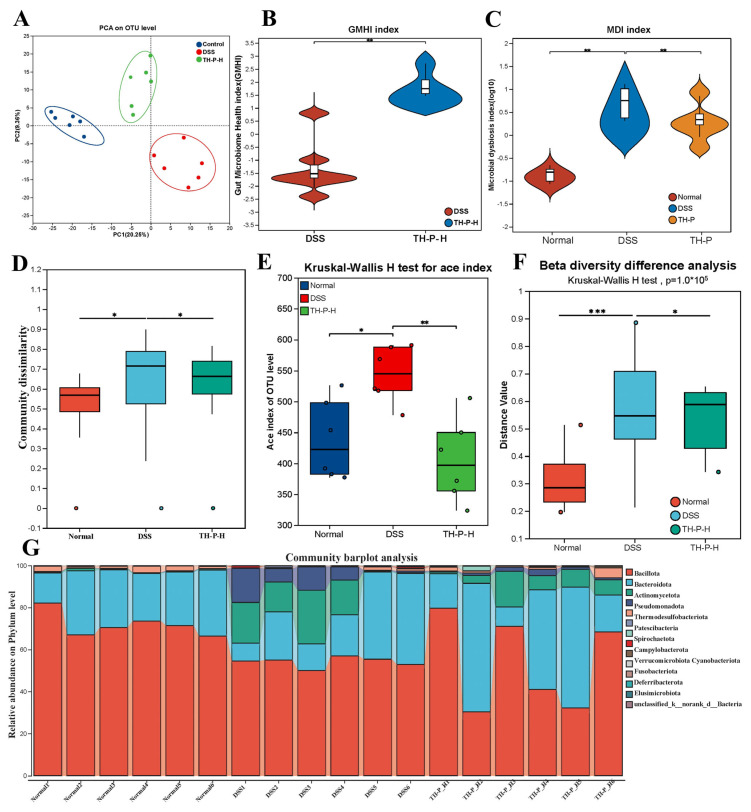
Gut microbiota analysis of mice with UC (*n* = 6). (**A**) PCA of microbial communities showing differences in TH-P, DSS and normal groups; (**B**) gut microbiome health index (GMHI) between TH-P and DSS groups; (**C**) microbiota dysbiosis index (MDI), (**D**) community dissimilarity, (**E**) ace index, (**F**) beta diversity difference analysis of TH-P, DSS and normal groups; (**G**) phylum-level composition of gut microbiota between TH-P group and DSS group. * *p* < 0.05, ** *p* < 0.01, *** *p* < 0.001 vs. DSS.

DSS markedly disrupted the gut microbiota structure, whereas TH-P restored the microbial community composition (Figure 8A). TH-P significantly improved the gut microbiome health index (GMHI) and microbial dysbiosis index (MDI) (Figure 8B,C). Microbiota-related indices (community dissimilarity, beta diversity difference analysis, and ace index from the Kruskal–Wallis H test) indicated improved intestinal health and stability following TH-P treatment (Figure 8D–F). Moreover, TH-P corrected phylum-level imbalances: TH-P increased the level of *Bacteroidota* and decreased the level of *Actinomycetota* and *Pseudomonadota* (Figure 8G). These results demonstrated that TH-P ameliorated the gut microbiota dysbiosis induced by DSS.

### 2.9. Metabolomics Analysis of Gut Contents of Mice with UC

**Figure 9:**
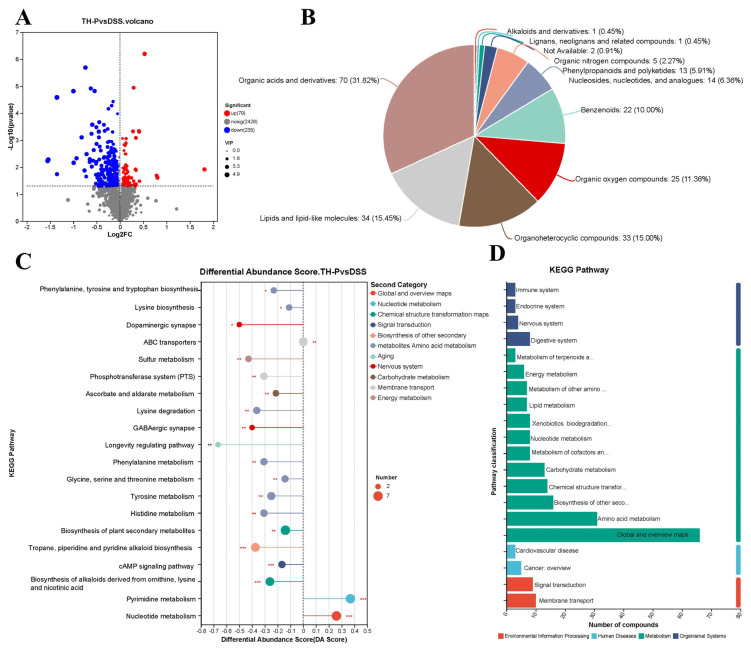
Gut content metabolomics analysis of mice with UC (*n* = 4). (**A**) Venn plot showing differential metabolites between TH-P group and DSS group; (**B**) metabolite classification, (**C**) key metabolites and (**D**) KEGG pathway enrichment between TH-P group and DSS group. * *p* < 0.05, ** *p* < 0.01, *** *p* < 0.001 vs. DSS.

TH-P markedly reversed DSS-induced alterations in intestinal metabolites (Figure 9A). Differential metabolites were enriched in organic acids and energy-metabolism-related pathways (Figure 9B). Key metabolites involved in host–microbiota interactions re shown in Figure 9C. Importantly, SCFA metabolism pathways were significantly enriched (Figure 9D). These findings demonstrated that TH-P improved intestinal metabolic profiles and promoted the production of organic acids and derivatives.

## 3. Discussion

UC is a chronic relapsing inflammatory disorder of the colon characterized by persistent mucosal inflammation, epithelial barrier disruption, and immune dysregulation [23]. Among the signaling networks implicated in UC pathogenesis, the PI3K/AKT pathway has emerged as a critical regulator of intestinal homeostasis [24]. This pathway is closely involved in epithelial cell survival [25], mucosal restitution, oxidative stress responses, and inflammatory control [26]. Increasing evidence indicates that impairment of PI3K/AKT signaling contributes to barrier dysfunction, exaggerated inflammatory responses, and progressive mucosal injury in colitis [27]. Conversely, the activation of this pathway has been reported to enhance the expression of tight junction proteins, improve epithelial repair, and limit intestinal inflammation [28]. Recent studies have also suggested that microbial metabolites, particularly short chain fatty acids, participate in the regulation of PI3K/AKT-dependent protective responses in the gut [29]. Therefore, targeting PI3K/AKT signaling has become an important mechanistic direction in the development of novel therapeutic strategies for UC.

Natural polysaccharides have attracted considerable attention as potential agents for the management of UC because of their broad bioactivities, low toxicity, and capacity for multilevel regulation [30,31]. A growing number of studies are showing that plant-derived polysaccharides can ameliorate experimental colitis through coordinated effects on oxidative stress, inflammatory mediator production, intestinal barrier integrity, gut microbiota composition, and microbial metabolite generation [32]. For example, polysaccharides isolated from *Dendrobium officinale*, *Astragalus membranaceus*, and *Polygonatum sibiricum* have been reported to attenuate DSS-induced colitis, with mechanisms involving microbiota remodeling, enhancement of short-chain fatty acid production, and modulation of host signaling pathways such as PI3K/AKT [33,34]. In contrast to conventional drugs that often target a limited number of pathways, polysaccharides may exert broader regulatory effects on both the intestinal microenvironment and host responses [35,36]. This multi-target mode of action is particularly advantageous in UC, a disease driven by the complex interaction of immune imbalance, oxidative injury, barrier dysfunction, and microbial dysbiosis [37].

*T. hemsleyanum* is a traditional medicinal plant with increasing pharmacological relevance, and its polysaccharide fraction has gradually gained attention as a major bioactive component [21]. Previous studies have demonstrated that polysaccharides from *T. hemsleyanum* possess antioxidant, anti-inflammatory, immunomodulatory, and tissue-protective activities [38]. Free radical scavenging, suppression of inflammatory cytokine release, and regulation of immune-related responses have all been described as important biological properties of this natural polysaccharide [39,40]. Based on the reported structure–function relationships [41], the antioxidant activity of TH-P can be explained by its compositional features. First, TH considerable -P contains amounts of uronic acids (19.61% glucuronic acid and 4.5% galacturonic acid), and the abundant carboxyl groups from these uronic acids are known to promote radical scavenging. Second, the polysaccharide has a relatively high proportion of galactose (15.18%) and a detectable level of arabinose (1.76%), both associated with enhanced DPPH and hydroxyl radical scavenging activities. Compared with our work, which primarily focused on the SOCS1/JAK2/STAT3 pathway and basic gut barrier restoration, the present study provides a substantial mechanistic advance by employing an integrated multi-omics strategy. For the first time, we revealed that TH-P alleviated UC through the activation of the PI3K/AKT signaling pathway, remodeling of glycerophospholipid and arachidonic acid metabolism, and specific modulation of the gut microbiota (enrichment of *Bacteroidota* and reduction in opportunistic *Actinomycetota* and *Pseudomonadota* [42]. The biological activities of TH-P are likely associated with its structural features, including molecular weight, monosaccharide composition, glycosidic linkages, and uronic acid content. However, despite these encouraging observations, the role of TH-P in UC and the molecular basis underlying its protective effects have not been fully clarified.

In this study, TH-P significantly alleviated DSS-induced colitis, as evidenced by the improved body weight loss, disease activity index, colon shortening, edema, and histopathological damage. TH-P also reduced ROS accumulation and pro-inflammatory cytokine production, consistent with its antioxidant and anti-inflammatory activities. Transcriptomic analysis revealed that TH-P reversed DSS-induced gene expression alterations, with enrichment of the PI3K/AKT pathway, which was further confirmed by Western blotting. Moreover, TH-P remodeled the gut microbiota by increasing beneficial Lactobacillus and reducing opportunistic pathogens such as Escherichia and Shigella, while serum metabolomics showed that TH-P corrected the disturbances in glycerophospholipid and arachidonic acid metabolism. Collectively, these findings indicate that TH-P attenuates UC through the coordinated regulation of oxidative stress, inflammatory responses, gut microbiota, microbial metabolites, and PI3K/AKT signaling.

These observations align with a growing body of evidence showing that the modulation of the gut microbiota structure and abundance can directly or indirectly influence PI3K/AKT pathway activity. The organic acids and derivatives are key metabolites generated by gut microbiota from dietary fiber and have been recognized as signaling molecules that regulate host physiology [43]. The organic acids and derivatives possess anti-inflammatory properties, and its oral or topical administration had been proposed as an adjunct to standard UC therapy. Notably, propionate exerted its protective effect by activating GPR43, which in turn inhibited NLRP3 inflammasome activation and downregulated the PI3K/AKT signaling pathway [44].

Based on these findings, TH-P offers several potential advantages as a candidate intervention for UC. First, its protective effect i not restricted to a single target but involves the simultaneous regulation of inflammatory, oxidative, microbial, and metabolic processes, which is highly related to the multifactorial nature of UC [44]. Second, TH-P acts on both host and microbial compartments, thereby providing broader regulation of the intestinal ecosystem and systemic metabolic homeostasis. Third, as a plant-derived natural polysaccharide, TH-P exhibits favorable safety and tolerability for long-term use. Future studies should focus on the detailed structure–activity relationship of TH-P and on clarifying the causal links among microbiota remodeling, organic acid and derivative production, and PI3K/AKT activation. Nevertheless, the present findings indicate that TH-P is a promising natural candidate for the prevention and management of UC.

## 4. Materials and Methods

### 4.1. Materials

*Tetrastigma hemsleyanum* Diels et Gilg was obtained from Wenzhou, Zhejiang Province (China). It was identified by the authors as the root of *T. hemsleyanum*, a plant of the genus *Tetrastigma* in the Vitaceae family. *T. hemsleyanum* was identified by one of the authors (Ling Zhang). The plant specimen (No. Zhang-20240918) was deposited at the Jiangxi University of TCM, Nanchang, China.

TH-P (62.35 g) was extracted by the water extraction and alcohol precipitation method. An appropriate amount of dried *T. hemsleyanum* (500 g) was weighed, crushed, and passed through a 60-mesh sieve. The dried powder was mixed with distilled water at a ratio of 1:25 (g/mL) and refluxed at 80 °C for 2 h each time (two extractions). The combined filtrate was concentrated to one-third of its original volume, followed by the addition of four volumes of 90% ethanol. After stirring for 5 min, the mixture was allowed to stand at 4 °C for 24 h. The supernatant was discarded after centrifugation, and the precipitate was collected. The precipitate was redissolved in water, deproteinized by the Sevag method, dialyzed, and freeze-dried to obtain the TH-P (TH-P yield was 12.47%). The total polysaccharide content in the TH-P was determined by the phenol-sulfuric acid colorimetric method and the m-hydroxydiphenyl method to be 90.23% ± 3.07% without endotoxin.

### 4.2. Characterization of TH-P

The molecular weight of TH-P was determined by high-performance gel permeation chromatography (HPGPC) using an Agilent (Santa Clara, CA, USA) 1260 system equipped with an evaporative light scattering detector (ELSD) and a TSK-GEL G4000PWXL column (7.8 × 300 mm). The sample was dissolved in ultrapure water (2 mg/mL), filtered through a 0.22 µm membrane, and eluted with 0.1 M NaNO_3_ at 0.5 mL/min. The molecular weight was calculated using a calibration curve of dextran standards.

The total polysaccharide content was determined by the phenol-sulfuric acid method using glucose as the standard. The uronic acid content was measured by the m-hydroxydiphenyl method with galacturonic acid as the reference. The total polyphenol content was assessed using the Folin–Ciocalteu method with gallic acid as the standard. The protein content was quantified by the Bradford method using bovine serum albumin as the standard.

For monosaccharide composition analysis, the polysaccharide was hydrolyzed with 2 M trifluoroacetic acid (TFA) at 120 °C for 2 h. The hydrolysate was evaporated to dryness, dissolved in water, and derivatized with 1-phenyl-3-methyl-5-pyrazolone (PMP). The derivatives were separated on a C_18_ column (250 × 4.6 mm, 5 µm) using a mobile phase of 0.1 M phosphate buffer (pH 6.8) and acetonitrile (83:17, *v*/*v*) at 1.0 mL/min, with detection at 250 nm. Monosaccharides were identified and quantified by comparison with standards.

Infrared spectra were recorded on a Fourier-transform infrared (FTIR) spectrometer (Thermo Nicolet™ Summit, NJ, USA). The sample (1–2 mg) was ground with KBr powder (100–200 mg) and pressed into a pellet. The spectrum was scanned in the range of 4000–400 cm^−1^ at a resolution of 4 cm^−1^.

### 4.3. In Vitro Cell Experiments

#### 4.3.1. Cell Culture

RAW 264.7 murine macrophages (CL-0190, Procell, Wuhan, China) were cultured in DMEM supplemented with 10% fetal bovine serum (FBS; A5256701, Gibco, Waltham, MA, USA) and 1% penicillin–streptomycin (BL505A, Biosharp, Shanghai, China) at 37 °C in a humidified incubator containing 5% CO_2_.

#### 4.3.2. In Vitro Antioxidant Activity Assay

The in vitro antioxidant activity of TH-P was evaluated using DPPH and ABTS radical scavenging assays. TH-P was tested at concentrations of 12.5, 25, 50, 100, 200, and 400 μg/mL, with ascorbic acid (VC) as a positive control. The assays were performed according to the manufacturer’s instructions (Beyotime, Shanghai, China). Absorbance was measured at 517 nm (DPPH) and 734 nm (ABTS) using a Multiskan™ FC microplate reader (Thermo, USA).

#### 4.3.3. Cell Viability Assay

RAW264.7 cells were seeded into 96-well plates at a density of 5 × 10^3^ cells per well. After 24 h of incubation, the cells were treated with various concentrations of TH-P (12.5, 25, 50, 100, 200, and 400 μg/mL) for an additional 24 h. Then, 10 μL of CCK-8 reagent (Beyotime) was added to each well and incubated for 2 h. Absorbance was measured at 450 nm using a Multiskan™ FC microplate reader (Thermo).

#### 4.3.4. ROS Generation Assay

RAW264.7 cells were seeded into 6-well plates at a density of 1 × 10^5^ cells per well and divided into the following groups: control, LPS alone (1 μg/mL), and LPS + TH-P (12.5, 25, 50, or 100 μg/mL). After 24 h of treatment, the cells were incubated with a ROS probe (10 μmol/L; Beyotime, S0033S) for 30 min at 37 °C. The cells were then washed with PBS, and intracellular ROS levels were measured using a BD FACSAria II flow cytometer (USA). Data were analyzed with CytExpert 2.4 software.

#### 4.3.5. Inflammatory Cytokine Assay

Cell grouping and treatments were consistent with those described in Section 4.3.2. After 24 h of incubation, cell culture supernatants were collected. The levels of nitric oxide (NO, Beyotime, S0020S), interleukin-6 (IL-6), interleukin-1β (IL-1β), and tumor necrosis factor-α (TNF-α) were measured using Thermo Fisher mouse ELISA kits (IL-6/88-7064-22, IL-1β/88-7013A-88, and TNF-α/88-7324-88).

#### 4.3.6. Quantitative Real-Time PCR (RT-qPCR) Analysis

Mouse colon tissue (20 mg) was placed in 200 μL of TRIzol reagent (Invitrogen, 15596018, Carlsbad, CA, USA) and homogenized three times at 50 Hz for 30 s each. The total RNA was extracted and reverse-transcribed into cDNA using a cDNA synthesis mix (Beyotime, D7182L, USA) according to the manufacturer’s protocol. Real-time PCR was performed using Universal SYBR Green Fast qPCR Mix (ABclonal, RK21203, Wuhan, China) on a StepOne Plus real-time PCR system (Applied Biosystems, USA). GAPDH was used as the internal control (Beyotime, QM00014S). The PCR cycling conditions were initial denaturation at 95 °C for 3 min, followed by 40 cycles of 95 °C for 5 s and 60 °C for 1 min. The primer sequences were as follows: IκBα (forward): 5′-GTTTCCCCTCATCTTTCCCTCA-3′, IκBα (reverse): 5′-GGGTGCGTCTTAGTGGTATCTGT-3′; NF-κb (forward): 5′-ATCGTGGAGCACTTGGTGACT-3′, NF-κb (reverse): 5′-GCCCTGGTAGGTT-ACTCTGTTGA-3′; TrκB (forward): 5′-TGAAACAAGCCACACACAG-3′, TrκB (reverse): 5′-AATCACCACCACGGCATAG-3′.

### 4.4. Animal Experiment Design

#### 4.4.1. Experimental Animals

Male C57BL/6J mice (6–8 weeks old, weighing 18–22 g) were used in this study. The animals were housed under specific-pathogen-free (SPF) conditions at 22 ± 2 °C with 50 ± 10% humidity under a 12 h light/dark cycle. Mice were allowed free access to food and water and were acclimatized for one week prior to the experiment. This study was approved by the Experimental Animal Ethics Committee of Nanchang Medical College (Approval No. NYLLSC20240419026).

#### 4.4.2. Grouping and Treatment

Sixty mice were randomly divided into six groups (*n* = 10 per group): control group, model group (DSS alone), DSS + low-dose TH-P (DSS + TH-P-L), DSS + medium-dose TH-P (DSS + TH-P-M), DSS + high-dose TH-P (DSS + TH-P-H), and DSS + mesalamine (5-ASA) as the positive control group. All DSS-treated groups received 2.5% (*w*/*v*) dextran sulfate sodium (DSS; Thermo, 88-7013A-88) in drinking water on days 0–7 and days 15–21, with normal drinking water on days 8–14. The control group had free access to normal water throughout the experiment. From day 8 to day 21, animals were administered the following treatments daily: control group, normal water + saline; DSS group, DSS cycle + saline; DSS + TH-P-L group, DSS cycle + 60 mg/kg TH-P; DSS + TH-P-M group, DSS cycle + 120 mg/kg TH-P; DSS + TH-P-H group, DSS cycle + 240 mg/kg TH-P; and DSS + 5-ASA group, DSS cycle + 50 mg/kg 5-ASA (013763500, Adamas life, Shanghai, China). The experiment lasted 21 consecutive days. Body weight, stool consistency, and fecal occult blood were recorded daily, and the disease activity index (DAI) was calculated using a standard scoring system.

#### 4.4.3. Sample Collection

At the end of the experiment, mice were fasted 12 h with free access to water and then anesthetized with sodium pentobarbital. Blood was collected from the orbital sinus and allowed to clot at room temperature for 30 min, followed by centrifugation at 3000 rpm for 15 min at 4 °C to obtain serum, which was stored at −80 °C. Colon tissues were excised, measured for length and weight, longitudinally opened, and rinsed with PBS. One portion was fixed in 4% paraformaldehyde for histopathological analysis, while the remaining tissue was snap-frozen in liquid nitrogen and stored at −80 °C. Cecal contents were also collected and stored at −80 °C.

### 4.5. Histopathological Examination

Colon tissues fixed in 4% paraformaldehyde were dehydrated, cleared, and embedded in paraffin. Sections were then prepared and stained with hematoxylin and eosin (H&E). Histological features, including mucosal integrity, crypt architecture, and inflammatory cell infiltration, were assessed under a light microscope, and pathological scores were assigned accordingly.

### 4.6. Transcriptomic Analysis

RNA extraction and sequencing: Colon tissues from the normal and DSS groups were collected for RNA-seq analysis (Shanghai Majorbio Biopharm Technology, Shangai, China). Total RNA was extracted, and mRNA was isolated using oligo(dT) magnetic beads (MedChemExpress, HY-K0228, Shanghai, China). The purified mRNA was fragmented, and first-strand and second-strand cDNA were synthesized. After purification, cDNA fragments were subjected to end repair, phosphorylation, and adapter ligation according to the library construction protocol. Libraries were size-selected for cDNA fragments of approximately 300 bp on 2% low-range ultra agarose, followed by PCR amplification (15 cycles) using Phusion DNA polymerase (NEB). The libraries were quantified using a Qubit 4.0 fluorometer and sequenced on a DNBSEQ-T7 platform (PE150) with the DNBSEQ-T7RS reagent kit (FCL PE150, version 3.0, BGI, China).

Transcriptomic data analysis: Sequencing reads were aligned to the mouse reference genome (GRCm38) using HISAT2. Gene expression levels were quantified with featureCounts. Differential expression analysis was performed using DESeq2, with thresholds of |log_2_| ≥ 1, and adjusted *p* < 0.05. KEGG pathway enrichment analysis was conducted using the ClusterProfiler package in R (v. 1.4.1717). Volcano plots, Venn diagrams, and heatmaps were generated using R.

### 4.7. Untargeted Metabolomics Analysis of Serum

Sample preparation: An aliquot of 100 μL serum was added to a 1.5 mL tube containing 800 μL of extraction solvent (acetonitrile: methanol, 1:1, *v*/*v*) spiked with four internal standards (including 0.02 mg/mL L-2-chlorophenylalanine). The mixture was vortexed for 30 s and sonicated at 5 °C and 40 kHz for 30 min. Proteins were precipitated by incubating at −20 °C for 30 min, followed by centrifugation at 13,000× *g* and 4 °C for 15 min. The supernatant was collected and dried under nitrogen. The residue was redissolved in 100 μL of acetonitrile: water (1:1, *v*/*v*), sonicated at 5 °C for 5 min, and centrifuged at 13,000× *g* and 4 °C for 10 min. The final supernatant was transferred to a vial for LC-MS/MS analysis.

LC-MS analysis: Metabolite analysis was performed using a UHPLC-Q-Exactive system (Orbitrap Exploris 240, Thermo) equipped with an ACQUITY UPLC HSS T3 column (Waters, Milford, MA, USA, 2.1 × 100 mm, 1.8 μm). Mobile phase A was water with 0.1% formic acid, and mobile phase B was acetonitrile with 0.1% formic acid. Data were acquired in both positive- and negative-ion modes.

Serum untargeted metabolomics data analysis: The preprocessed data matrix was uploaded to the Majorbio Cloud Platform (cloud.majorbio.com). The variables with missing values in >80% of the samples were removed; the remaining missing values were imputed with the minimum value from the original matrix. Peak intensities were normalized using the sum normalization method to correct for instrument instability. Variables with a relative standard deviation (RSD) > 30% in quality control (QC) samples were removed, and the data were log10-transformed to obtain the final data matrix.

Principal component analysis (PCA) and orthogonal partial least squares discriminant analysis (OPLS-DA) were performed using the ropls package (version 1.6.2) in R, with seven-fold cross-validation to assess model stability. Metabolites with variable importance in projection (VIP) > 1 and Student’s *t*-test *p* < 0.05 were considered significantly different. These metabolites were annotated using the KEGG database (https://www.kegg.jp/kegg/pathway.html (accessed on 17 March 2024)). Pathway enrichment analysis was performed using Fisher’s exact test (scipy.stats package in Python, v 8.5.7) to identify the biological pathways most strongly associated with the experimental treatments.

### 4.8. Gut Microbiota Analysis

Total microbial genomic DNA was extracted from normal, DSS-treated, and TH-P-treated mouse colon samples using a FastPure Stool DNA Isolation Kit (MJYH, Shanghai, China) according to the manufacturer’s instructions. DNA quality and concentration were assessed by 1.0% agarose gel electrophoresis and a NanoDrop2000 spectrophotometer (Thermo, USA), and the DNA was stored at −80 °C until use. The V3–V4 hypervariable region of the bacterial 16S rRNA gene was amplified using primers 338F (5′-ACTCCTACGGGAGGCAGCAG-3′) and 806R (5′-GGACTACHVGGGTWTCTAAT-3′) on a T100 Thermal Cycler (Bio-Rad, Hercules, CA, USA). The PCR reaction mixture (20 μL) contained 4 μL of 5× FastPfu buffer, 2 μL of 2.5 mM dNTPs, 0.8 μL of each primer (5 μM), 0.4 μL of FastPfu polymerase, 10 ng of template DNA, and ddH_2_O to volume. The cycling conditions were an initial denaturation at 95 °C for 3 min; 27 cycles of 95 °C for 30 s, 55 °C for 30 s, and 72 °C for 45 s; and a final extension at 72 °C for 10 min. The PCR product was extracted from 2% agarose gel, purified with a PCR Clean-Up Kit (YuHua, Shanghai, China), and quantified using a Qubit 4.0 fluorometer (Thermo).

Gut microbiota data analysis: Raw sequencing data were processed using QIIME 2. Amplicon sequence variants (ASVs) were generated, and taxonomic annotation was performed using the Silva database. Alpha diversity indices (Shannon, Chao1, and Ace) and beta diversity (principal component analysis based on Bray–Curtis distance) were calculated. LEfSe analysis (linear discriminant analysis effect size; LDA score > 2) was used to identify differentially abundant taxa.

### 4.9. Western Blot Analysis

Colon tissues were lysed in RIPA buffer (Beyotime, P0013C) supplemented with a protease and phosphatase inhibitor cocktail (Beyotime, P1045). Protein concentrations were determined using the BCA method. Equal amounts of protein (30 μg) were separated by SDS-PAGE and transferred onto PVDF membranes. The membranes were blocked with 5% non-fat milk for 1 h and then incubated overnight at 4 °C with primary antibodies against GAPDH (Beyotime, AF1186), PI3K (ABclonal, A22730), and AKT (Beyotime, AA326). After washing, the membranes were incubated with HRP-conjugated secondary antibodies for 1 h at room temperature. Protein bands were visualized using an ECL detection system and quantified using ImageJ 1.8.0 software, with GAPDH as the internal control.

### 4.10. Statistical Analysis

Data were analyzed using GraphPad Prism 8.0. Results are presented as mean ± standard deviation (SD). Comparisons among multiple groups were performed using one-way ANOVA followed by Tukey’s post hoc test. *p* < 0.05 was considered statistically significant.

## 5. Conclusions

In this study, a polysaccharide (TH-P) isolated from *T. hemsleyanum* was systematically investigated, and its protective effect against UC was elucidated using a multi-omics approach. TH-P exhibited notable antioxidant and anti-inflammatory activities and effectively alleviated DSS-induced colitis by improving clinical symptoms and restoring intestinal barrier integrity. Mechanistically, TH-P exerted protective effects by modulating multiple pathways. It regulated inflammatory cytokine production, reshaped the gut microbiota by enriching beneficial taxa (*Lactobacillus*) while reducing opportunistic pathogens (*Escherichia* and *Shigella*). Multi-omics integration further linked these effects to the regulation of glycerophospholipid and arachidonic acid metabolism, alongside the activation of the PI3K/AKT signaling pathway. In conclusion, these findings demonstrate that TH-P alleviates UC via multi-target mechanisms and highlight its potential as a functional food ingredient or adjunct therapeutic agent for intestinal health.

## Data Availability

Data will be provided upon request.

## Supplementary Materials

The following supporting information can be downloaded at: https://www.mdpi.com/article/10.3390/molecules31111974/s1, Figure S1: Chromatogram plot and the standard of HPGPC about TH-P; Figure S2: Distribution plot of HPGPC about TH-P; Figure S3: The standard of monosaccharide composition about TH-P; Table S1: The molecular parameters of TH-P.
