# Supplementary material for: Mechanism Through Which Antioxidant Polysaccharide from Tetrastigma hemsleyanum Protects Against DSS-Induced Ulcerative Colitis: Insights from Multi-Omics
Source: Molecules. 2026 Jun 5;31(11):1974. doi: 10.3390/molecules31111974 (PMC13258601; doi:10.3390/molecules31111974)
Supplement: Supplementary file 1 [file molecules-31-01974-s001.zip › molecules-4283026-supplementary.pdf]

Table S1. The molecular parameters of TH-P

	Mp (g/mol)	Mn (g/mol)	Mw (g/mol)	Mz (g/mol)	Mz+1 (g/mol)	Mv (g/mol)	PD
TH-P	53221	39617	55474	84869	1099962	81124	1.47121

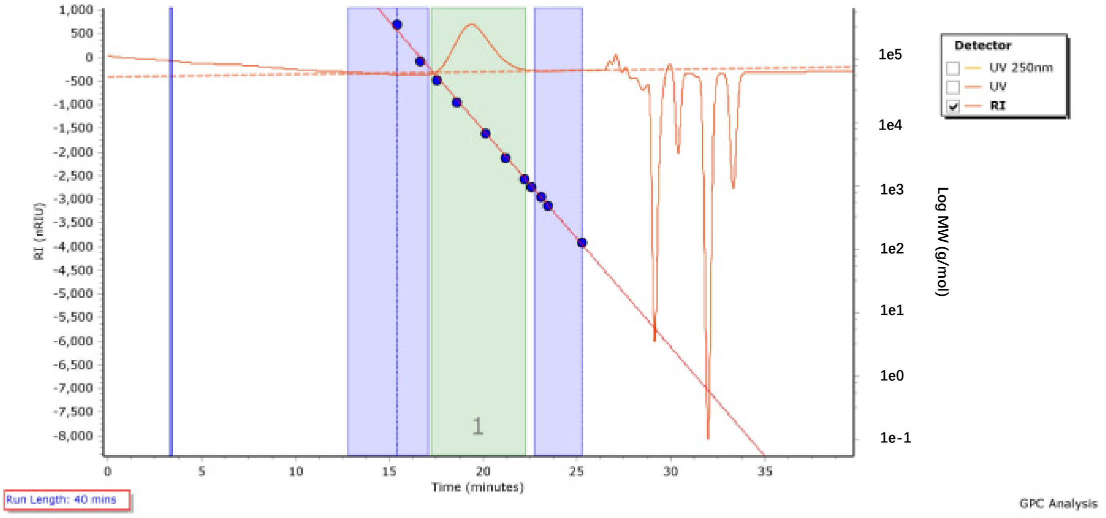

Figure S1. Chromatogram plot and the standard of HPGPC about TH-P

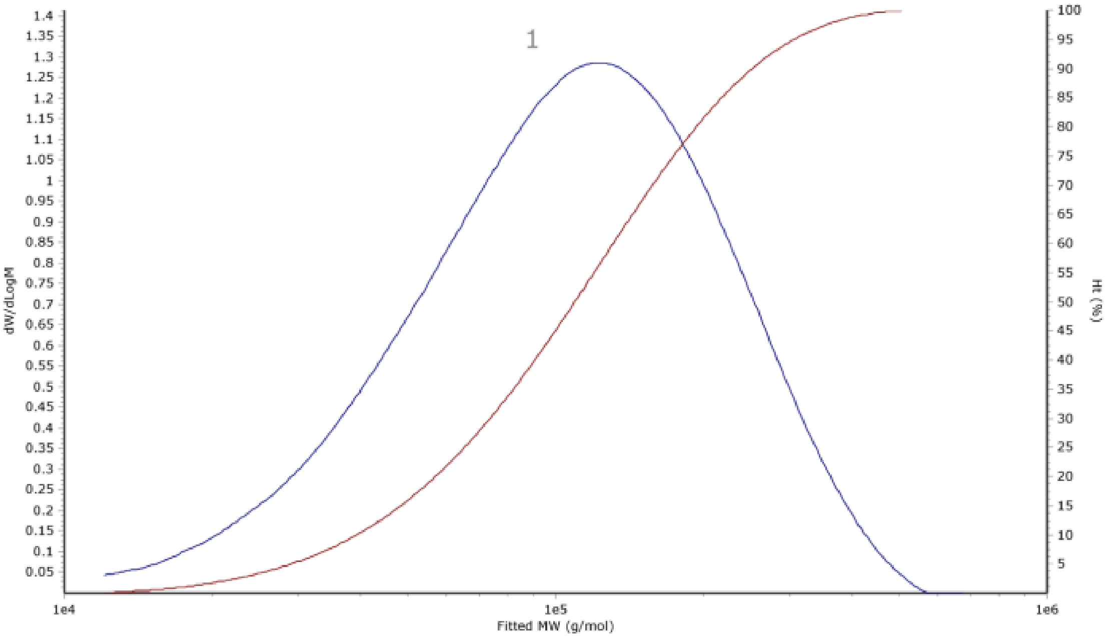

Figure S2. Distribution plot of HPGPC about TH-P

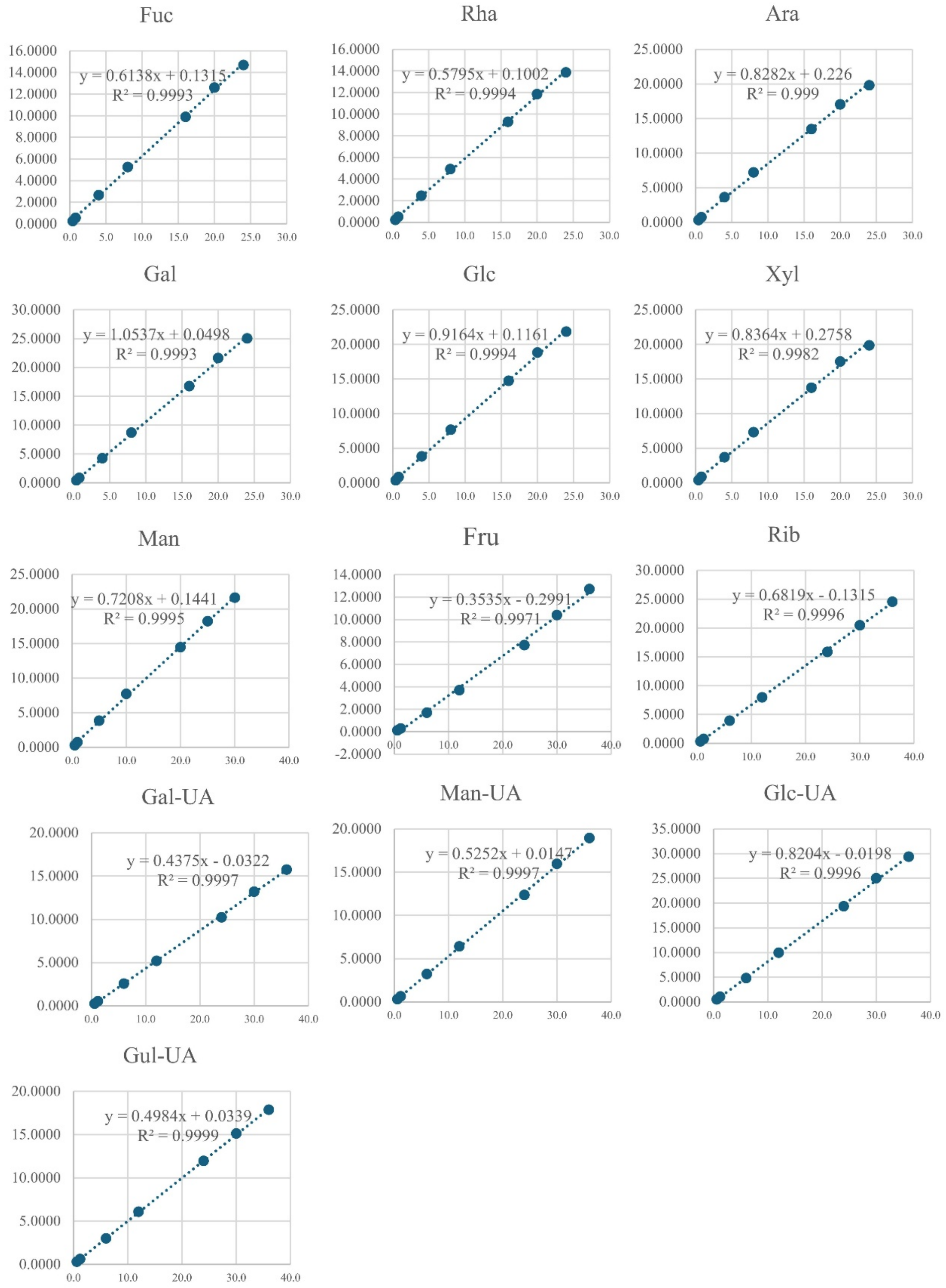

Figure S3. The standard of monosaccharide composition about TH-P
